# Recent Research Progress of Chiral Small Molecular Antitumor-Targeted Drugs Approved by the FDA From 2011 to 2019

**DOI:** 10.3389/fonc.2021.785855

**Published:** 2021-12-17

**Authors:** Xuetong Chu, Yizhi Bu, Xiaoping Yang

**Affiliations:** Key Laboratory of Study and Discovery of Small Targeted Molecules of Hunan Province, Department of Pharmacy, School of Medicine, Hunan Normal University, Changsha, China

**Keywords:** chiral, small molecule, antitumor-targeted drugs, recent research, FDA

## Abstract

Chiral drugs usually contain chiral centers, which are present as single enantiomers or racemates. Compared with achiral drugs, they have significant advantages in safety and efficacy with high stereoselectivity. Of these drugs, chirality not only exerts influence on the solubility and pharmacokinetic characteristics but also has specific mechanistic characteristics on their targets. We noted that small molecules with unique chiral properties have emerged as novel components of antitumor drugs approved by the FDA in decade. Since approved, these drugs have been continuously explored for new indications, new mechanisms, and novel combinations. In this mini review, recent research progress of twenty-two FDA-approved chiral small molecular-targeted antitumor drugs from 2011 to 2019 is summarized with highlighting the potential and advantages of their applications. We believe that these updated achievements may provide theoretical foundation and stimulate research interests for optimizing drug efficacy, expanding clinical application, overcoming drug resistance, and advancing safety in future clinical administrations of these chiral targeted drugs.

## 1 Introduction

Chiral molecules first were found in 1848, stemed from the origin of stereochemistry (1). So far, chiral compounds have covered various fields including chemical materials and pharmaceutical industry. In recent years, chiral drugs have gradually become the focus of new drug development and clinical application due to their unique profiles. Chiral small molecular targeted tumor drugs have good target binding characteristics origined from their high stereoselectivity, which can reduce the entry of inactive drugs or low-activity drugs and reduce toxic and side effects simutanuously. Thus, chiral small molecular drugs have obvious advantages and broad development prospects in antitumor-targeted therapy.

Small molecular targeted drug therapy provides more choices and survival opportunities for cancer patients who are resistant to chemotherapy, but drug resistance and safety also plague this treatment method. It is valuable to discuss the role of molecular chirality in efficacy, safety, and drug resistance from the structural characteristics of small molecular targeted drugs. Undoubtedly, the study of chiral structure will give more detailed explanation and supplement to the mechanism of drug action and provide reliable reference for the research and development of new drugs.

Chiral small molecular targeted anticancer drugs have become a novel component for patient treatments with twenty-two drugs approved by the FDA from 2011 to 2019. Since approved, these drugs have demonstrated their excellent therapeutic effects on patients clinically. In the meantime, their new mechanisms, new indications, and novel combination regimens have been actively explored. For example, drugs approved for the treatment of malignant hematological tumors have shown reliable efficacy in the treatment of solid tumors. Moreover, the results from either *in vitro* or *in vivo* expanded new mechanisms of action. Moving forward, the combination of chiral small molecular targeted drugs with other drugs including monoclonal antibodies shows super anticancer efficancy and safety. Profoundly, strategies to overcome drug resistance by applying these chiral targeted drugs have made a solid progress recently. Saha et al. (2) summarized the critical role of chirality on the improvement of druggability within human kinome. They pointed out that chiral kinase inhibitors have controllable and positive effects on pharmacokinetics. Chiral centers in drug molecules have specific characteristics in chiral environment, and the direct modification can optimize the efficacy of drug molecules. However, they did not focus on the recent progress of the identification of new indications, or exploration of new mechanisms of action.

**Figure 1:**
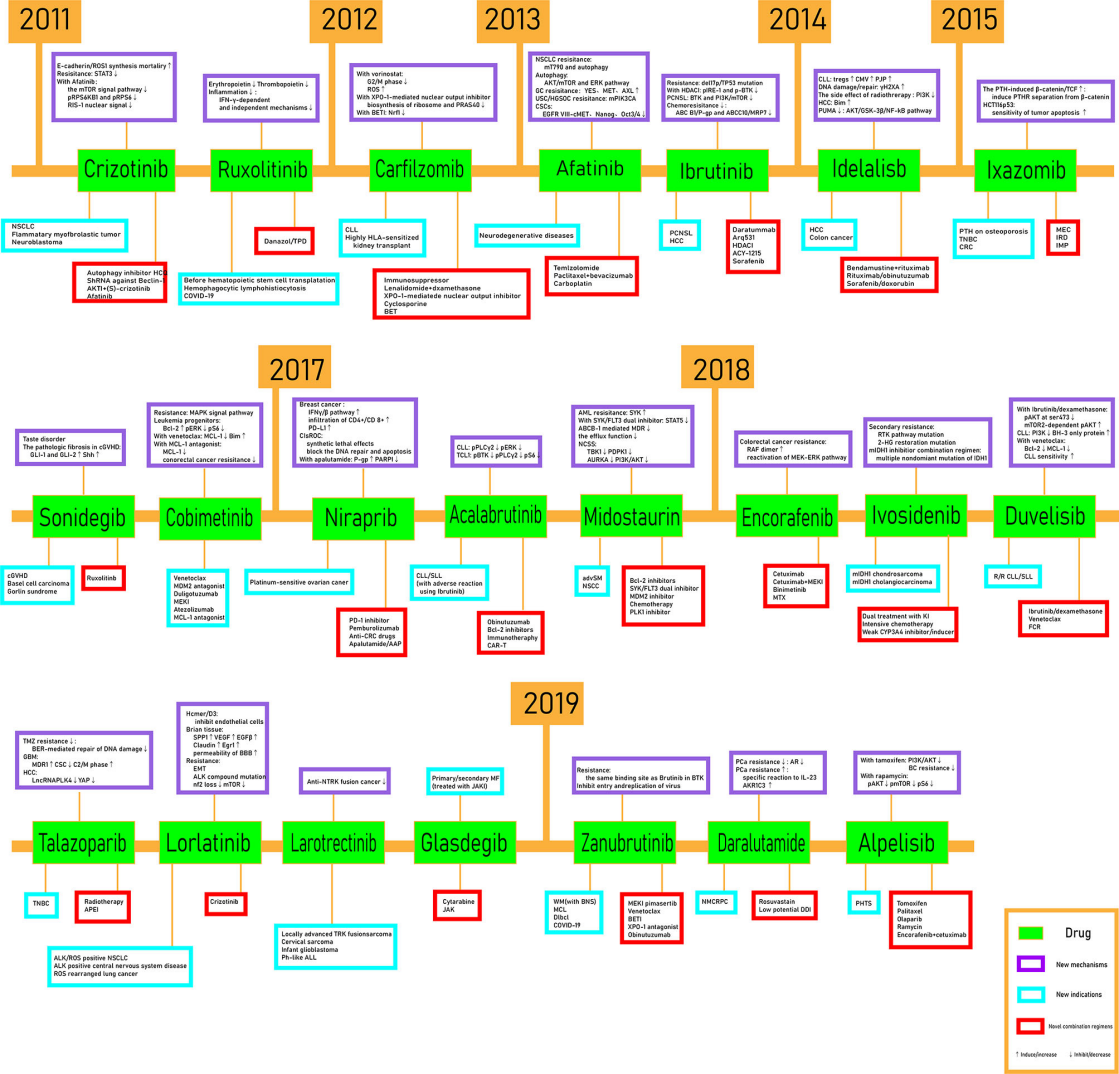
The schematic figure.

**Figure 2:**
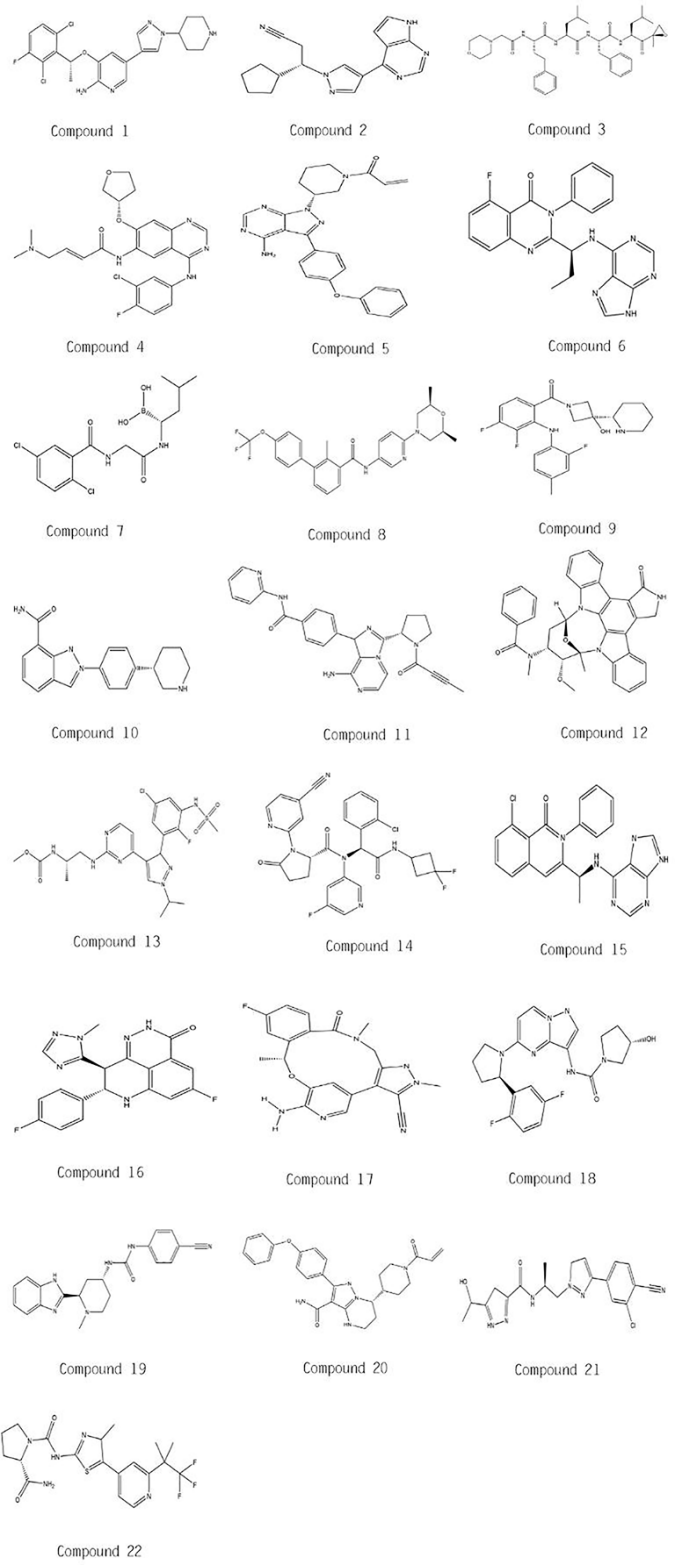
Molecular chemical structures of twenty-two drugs.

In this mini review, recent clinical-related progress of new mechanisms, new indications, and novel combination regimens of twenty-two chiral small molecular antitumor-targeted drug approved by the FDA from 2011 to 2019 is summarized. The role of the chiral characteristics of these drugs will be emphasized. The advantages and disadvantages of chiral drugs will be comprehensively evaluated. At the very beginning, the basic profiles of twenty-two chiral small molecular targeted antitumor drugs are comprehensively summarized as **Table 1**. The summary diagram of this review is shown in **Figure 1**. The molecular structural formulas of twenty-two drugs are posted in **Figure 2**. Detailed information will then be presented in sequential years. We wish that the current work will establish an accurate and detailed approach for optimizing the efficacy of chiral drugs, expanding their clinical application, overcoming drug resistance and improving safety, and also provide a reliable fashion for the research of chiral drug active enantiomers.

**Table 1:** Basic profiles of twenty-two chiral small molecular targeted antitumor drugs.

Compound serial number	Drugs	Trade name	Original research company	Target spot	FDA approved the time of listing	FDA approved the indications
1	Crizotinib	Xalkori	Pfizer	ALK, c-Met, ROS1, RON	August 26, 2011	August 26, 2011—NSCLC
						March 11, 2016—NSCLC with ROS1 mutated
2	Ruxolitinib	Jakav	Incyte	JAK1/2 kinase	November 16, 2011	November 16, 2011—IMF, PPMF, PPV-MF
						December 4, 2014–PV
						April 24, 2019—aGVHD
3	Carfilzomib	Kyprolis	Onyx	N-terminal threonine active site of 20S protease	July 20, 2012	July 20, 2012—refractory MM
4	Afatinib	Gilotrif	Boehringer-Ingelheim	ErbB1/2/4	July 17, 2013	July 17, 2013—NSCLC of missing EGFR exon 19 or the alternative mutation of exon 21
						April 15, 2016—metastatic NSCLC of nonresistant rare EGFR mutations
5	Ibrutinib	Imbruvica	Johnson & Johnson and Pharmacyclics	The active site Cys-481 of BTK	November 13, 2013	November 13, 2013—MCL
						February 12, 2014—CLL
						July 28, 2014—CLL carrying del 17p deletion mutations
						January 29, 2015—WM
						March 4, 2016—first-line treatment of CLL
						May 6, 2016—benendamostetin + rituximab (BR) for the treatment of SLL
						January 19, 2017—MZL
						August 27, 2018—combined with rituximab for the treatment of LPL
						August 3, 2017—cGVHD
						January 28, 2019—combined with otuzumab (Gazyva) for treating adult patients with newly diagnosed CLL/SLL
						April 21, 2020—combined with rituximab for initial treatment in adult patients with CLL/SLL
6	Idelalisib	Zydelig	Gilead science	PI3K	July 23, 2014	July 23, 2014—FL/SLL/in conjunction with rituximab for the treatment of CLL
7	Ixazomib	Ninlaro	Takeda Pharmaceutical	The β5 subunit of 20S proteasomes	November 29, 2015	November 29, 2015—in combination with lenalidomide and dexamethasone for patients with MM
8	Sonidegib	Odomzo	Novartis, Switzerland	SMO	July 24, 2015	July 24, 2015—local advanced basal cell cancer that has recurred after or is not suitable for surgery or radiotherapy
9	Cobimetinib	Cotellic	Roche’s Genentech	MEK	November 10, 2015	November 10, 2015—atezolizumab + cobimetinib + vemurafenib for the joint treatment of late melanoma with BRAF V600 mutations
10	Niraparib	Zejula	TESARO	PARP-1/2	March 27, 2017	March 27, 2017—maintenance treatment for patients with recurrent ovarian, tubal, or primary peritoneal cancer completely or partially relieved after platinum chemotherapy
						October 23, 2019—advanced ovarian, tubal, or primary peritoneal cancer (carrying PARP mutations) that have received 3 or more chemotherapy options
						April 29, 2020—first-line maintenance treatment (whether or not PARP mutations) in patients with advanced ovarian, tubal, or primary peritoneal cancer after first-line platinum chemotherapy
11	Acalabrutinib	Calquence	AstraZeneca	BTK	October 31, 2017	October 31, 2017—CLL/SLL
12	Midostaurin	Rydapt	Novartis	Protein kinase C α (PKC α)	April 28, 2017	April 28, 2017—ASM/SM-AHN/MCL
13	Encorafenib	Braftovi	Novartis	BRAF	June 27, 2018	June 27, 2018——unresectable or metastatic melanoma patients with BRAF V600E or BRAF V600K mutation confirmed
						April 8, 2020—mCRC with BRAF V600E mutation
14	Ivosidenib	Tibsovo	Agios Pharmaceuticals	Mutant IDH1	July 20, 2018	July 20, 2018—R/R AML in human with IDH1 mutation
						May 2, 2019—AML patients aged 75 and over who could not use intensive chemotherapy due to other complications
15	Duvelisib	Copiktra	Verastem	PI3K	September 24, 2018	September 24, 2018—R/R CLL/SLL/FL
16	Talazoparib	Talzenna	Pfizer	PARP	October 17, 2018	October 17, 2018—locally advanced or metastatic breast cancer with BRCA mutation (harmful or suspected harmful) and HER2 negative
17	Lorlatinib	Lorbrena	Pfizer	ALK	November 2, 2018	November 2, 2018—ALK-positive metastatic NSCLC
18	Larotrectinib	Vitrakvi	Bayer and Loxo Oncology	TRKs	November 26, 2018	November 26, 2018—adult and child patients with locally advanced or metastatic solid tumors with NTRK gene fusion
19	Glasdegib	Daurismo	Pfizer	SMO	November 2, 2018	November 2, 2018—— in combination with low-dose cytarabine for the treatment of untreated AML
20	Zanubrutinib	Brukinsa	Baekje Shenzhou	BTK	November 15, 2019	November 15, 2019—R/R MCL
21	Darolutamide	Nubeqa	Bayer Pharmaceuticals	Androgen receptor	July 30, 2019	July 30, 2019—NM-CRPC
22	Alpelisib	Piqray	Novartis	PI3K	May 4, 2019	May 4, 2019—combined with Fulvestrant in the treatment of advanced metastatic breast cancer with hormone receptor positive (HR+)/human epidermal growth factor receptor 2 negative (HER2−) and PIK3CA mutation in male and postmenopausal women

## 2 The Chiral Small Molecular Targeted Antitumor Drugs Approved by the FDA in 2011

Crizotinib (1) and ruxolitinib (2) were approved by the FDA for listing in 2011, the former for nonsmall cell lung cancer (NSCLC) caused by ALK/ROS1 mutation and the latter for idiopathic myelofibrosis, postpolycythemic myelofibrosis, postpolycythemia vera myelofibrosis, polycythemia vera, and acute graft-versus-host disease. In addition, their approved chiral configuration for listing by the FDA is (R)-enantiomer.

### 2.1 Crizotinib

#### 2.1.1 New Mechanisms

Crizotinib can induce the increase of E-cadherin/ROS1 synthesis mortality, leading to abnormal mitosis and multinucleation in cells with E-cadherin deficiency. The phenotype is related to cytoplasmic division deficiency and abnormal phosphorylation and localization of p120 catenin (3). In the meantime, crizotinib affects other RTK activities and makes the range of downstream targets wider (4, 5). On drug resistance, crizotinib inhibited cytoplasmic STAT3, led to EIF2A phosphorylation, then inhibited nuclear STAT3, and then downregulated B-cell lymphoma gene-2 (BCL-2), and finally led to a high level of protective autophagy in lung cancer cells (6). In addition, combined with afatinib, crizotinib inhibits the mTOR/insulin signaling pathway and downgrades the pRPS6KB1 and pRPS6 downstream, and then cake-specific elimination of IRS-1 nuclear signals in the signaling pathway (7).

As for (S)-crizotinib, it has the targeted inhibition of MTH1, which can destroy the homeostasis of nucleotide library, induce the increase of DNA single-strand breaks, activate DNA repair of human colon cancer cells, and effectively inhibit tumor growth in animal models. The theoretical support for the stereospecificity of (S)-enantiomer was obtained by enzyme activity assay, chemical proteomic analysis, kininome activity assay, and MTH1 eutectic structure (8). In addition, (S)-crizotinib inhibits gastric cancer cell growth through oxidative DNA damage mechanism and can trigger survival promoting AKT signal at the same time, which increase the growth rate γ-H2AX and Ser1981 phosphorylated ataxia telangiectasia-mutated gene, while *N*-acetyl-l-cysteine could inhibit this effect of (S)-crizotinib. The inhibition of activated AKT will enhance the inhibitory effect of (S)-crizotinib on the growth of gastric cancer tumor cells and resensitize them (9).

It is worth noting that the two also have a certain inhibitory effect on each other’s target points. (S)-crizotinib induced NSCLC cell apoptosis by increasing ROS and activating endoplasmic reticulum stress pathway, and the whole process was independent of MTH-1 (9). Thus, we can speculate that there may be an interconnection between the two inhibitory pathways.

#### 2.1.2 New Indications

Compared with single-dose chemotherapy (pemetrexed or docetaxel), crizotinib has better therapeutic effect on NSCLC (10, 11). The satisfactory therapeutic effects of crizotinib can be observed by treating inflammatory myofbroblastic tumors with ALK rearrangement (12, 13) and neuroblastoma with R1275Q mutation in ALK (14).

Crizotinib has potential for breast cancer with E- cadherin deficiency (3). The treatment of alveolar rhabdomyosarooma prospectively benefits from crizotinib which better solve the drug resistance during chemotherapy and radiotherapy (4, 5).

#### 2.1.3 Novel Combination Regimens

The study of animal model found that autophagy inhibitor HCQ, drug inhibitor, or shRNAs against Beclin-1 can enhance the antitumor activity of crizotinib (6). In addition, the combination of AKT inhibition and (S)-crizotinib has the potential to become a new clinical scheme for the treatment of gastric cancer (9). Beyond that, the combination of afatinib and crizotinib hopefully becomes a new treatment of disseminated cutaneous malignant melanoma (7).

### 2.2 Ruxolitinib

#### 2.2.1 New Mechanisms

JAK2 has preferential selectivity to (R)-enantiomers of ruxolitinib and its aniline derivative. Their (S)-enantiomers adjust to JAK2 by rotating, which still does not fit well. This demonstrates that the chirality center is the key to a combination with JAK2. Similarly, the achiral analog barcitinib shows the same conformation as ruxolitinib in the binding mode with JAK2. In a word, the structure-activity relationship of ruxolitinib is supposed to be a core in the study of curative effect. On account of the high selectivity of ruxolitinib for JAK2, patients whose diseases caused by JAK2 mutation may benefit more from ruxolitinib combination therapy, which encourages that gene mutation detection is applied to patients.

Ruxolitinib plays a momentous part in signal transduction of erythropoietin and thrombopoietin, thus ruxolitinib can cause dose-dependent anemia and thrombocytopenia, which can be predictable (15, 16). In addition, the dose is adjusted on the basis of the actual situation when patients have kidney function damage (17). Ruxolitinib passes through IFN-γ-dependent and IFN-γ-independent mechanisms inhibiting inflammation, activation, and tissue infiltration of T cells and some undetected neutrophils (18).

#### 2.2.2 New Indications

The use of ruxolitinib before hematopoietic stem cell transplantation can improve the success rate of transplantation (19).

The preliminary clinical research data (20) show that ruxolitinib is positive, well tolerated and controllable in the treatment of five hemophagocytic lymphohistiocytosis patients with secondary hemophagocytic lymphohistiocytosis. In addition, ruxolitinib can participate in the immune regulation of COVID-19 patients and reduce symptoms, but the research results did not show that ruxolitinib can reduce virus (21, 22).

#### 2.2.3 Novel Combination Regimens

There are some debates in the effect of ruxolitinib on prolonging the survival of patients with myelofibrosis (23). It seems that novel combination regimens of ruxolitinib are a reliable approach to optimizing both efficacy and safety. The favorable efficacy and safety of the combination have been proved. In addition, ruxolitinib combined with danazol or TPD is a good therapy in elevating hemoglobin (Hgb) and platelets (PLT) (23, 24).

## 3 The Chiral Small Molecular Targeted Antitumor Drug Approved by the FDA in 2012

Carfilzomib (3) was approved by the FDA for listing in 2012, for patients with multiple myeloma who had received at least 2 drugs prior to treatment, which contains five chiral centers.

### 3.1 Carfilzomib

#### 3.1.1 New Mechanisms

Carfilzomib has the less nontargeted effects so as to exert lower neurotoxicity and higher safety, as the second proteasal inhibitor approved by the FDA (25–27). In addition, vorinostat and the histone deacetylase inhibitor both inhibit phase G2/M, while increasing ROS level in the cellular environment, and mitogen-activated protein kinase (MAPK), such as stress activase JNK, p38MAPK, which also promotes the lethality of carfilzomib and vorinostat to cancer cell. Also, there is likely an amplification loop between ROS and p38MAPK (28). Combined with XPO1-mediated nuclear output inhibitors, carfilzomib could interfere with the biosynthesis of ribosomes and inhibit survival-promoting kinase PRAS40, and then achieve anticancer effects. Beyond that, in virtue of BET inhibitors, the ability of transcription factor Nrf1 to induce proteasal genes to proteasal inhibition is weakened, hindering the rebound reaction of proteasal activity, which is the key pathway for cells to address protein toxic stress. BET inhibitors have the potential to combine with carfilzomib to treat solid tumors (29).

In safety, carfilzomib causes the myocardial activation of PP2A, then deactivates AMPKαand the downstream signaling related to autophagy, and results in the acute cardiac dysfunction. Metformin can offer the cardiprotection by recovering the phosphorylation of AMPKα. It is also speculated that nephrotoxicity may be caused by the potential effects of carfilzomib on renal endothelial cells, or may have similar pathologic mechanisms to cardiovascular toxicity (30).

#### 3.2.2 New Indications

Carfilzomib targets more selectively the chymotrypsin-like activity of the proteasome than bortezomib, which has been observed to have a certain therapeutic effect on bortezomib resistance cells and patients *in vivo* and *in vitro* (31–33). Also, carfilzomib can be used in the treatment of chronic lymphacytic leukemia, and a heterogeneous response and a variability have been observed among patients (34). In the meantime, carfilzomib as a candidate in highly HLA-sensitized kidney transplant showed good safety and lower toxicity, and significantly reduced levels in bone marrow plasma cells and anti-HLA antibodies (35).

#### 3.2.3 Novel Combination Regimens

Favorable activity and safety have been observed in the treatment of newly diagnosed multiple myeloma (NNMM) applying carfilzomib combined with immunosuppressor. The new combination regimen of the three drugs, carfilzomib, lenalidomide, and dexamethasone, will be highly valuable (36). In addition, carfilzomib combined with XPO1-mediated nuclear output inhibitors can show up a satisfactory effect for the treatment of adiposarcoma. High-throughput screening points out that the combination of carfilzomib and cyclosporine has the potential to resisit adiposarcoma (37). Importantly, a synergy between carfilzomib and bromodomain extra-terminal (BET) family protein inhibitors can be used jointly for a variety of solid tumors (29).

## 4 The Chiral Small Molecular Targeted Antitumor Drugs Approved by the FDA in 2013

Afatinib (4) and ibrutinib (5) were approved by the FDA to its list in 2013, the former for the metastatic NSCLC of missing EGFR exon 19 or the alternative mutation of exon 21 or nonresistant rare EGFR mutations (L861Q, G719X, S768I), and the latter for mantle cell lymphoma, chronic lymphocytic leukemia, fahrenheit giant globinemia, small lymphocytic lymphoma (as monotherapy or combined with benendamostetin + rituximab), recurrent refractory borderline lymphoma, chronic graft antihost disease, and rare lymphoplasmic cell lymphoma (combined with rituximab). (S)-Afatinib and (R)-ibrutinib are the active enantiomers.

### 4.1 Afatinib

#### 4.1.1 New Mechanisms

T790M mutation and autophagy are perceived as the main mechanism that results in the resisitance of EGFR-mutant advanced NSCLC when using the tyrosine kinase inhibitor, afatinib. *In vitro*, afatinib induces the autophagy, which can show higher cytotoxicity when the autophagy was suppressed by chloroquine (CQ) and 3-MA. Futhermore, the autophagy induced by afatinib needs the paticipation of Akt/mTOR and Erk signaling pathways and ROS. The results of studies *in vivo* are the same (38, 39). A naphquinone compound shikonin plays a good role in inducing apoptosis and has a negative regulation on the PI3K/AKT signaling pathway, which is also considered a possible mechanism for shikonin with anti-NSCLC activity (40).

Afatinib induces the activation of MET and AXL in HER2-driven resistant gastric cancer cell lines, which can be inhibited by cabozantinib so as to reduce resisitance. YES1, a member of the Src family, is inhibited by dasatinib so that the resisitance is attenuated. Obviously, YES1, MET, and AXL activation in HER2-driven gastric cancer cells is a new mechanism for producing resistance, while the use of the corresponding activation inhibitor is a reliable method (41).

Abnormal expression of gene products commonly caused by amplification/mutation of HER2 and PIK3CA in high-grade serum endometrial cancer (USC) and ovarian cancer (HGSOC) and PIK3CA mutations are likely results of afatinib resistance, which can be overcome by HER2 combined with PIK3CA, AKT, or MTOR inhibitors. Also, the examination of PIK3CA/PIK3R1 carcincinogenic mutations may be the basis for determining whether the patient has afatinib single treatment resistance (42).

EGFRvIII/AKT, EGFRvIII/JAK2/STAT3, and focal adhesion kinase (FAK) are closely related with afatinib playing anticancer roles. EGFRvIII-cMET crosstalk in chemoradiation-resistant cancer stem cells (CSCs) is specifically inhibited by afatinib, thus inhibiting the expression of Nanog and Oct3/4 as well. *In vitro* experiments show that afatinib combined with temozolomide can inhibit the self-renewal properties of Nanog and Oct3/4 (43). In addition, afatinib can block the phosphorylation of EGFR, AKT, and ERK caused by oxygen/glucose deprivation (OGD), thereby preventing the activation of subsequent pathways. Moreover, afatinib attenuated OGD-induced astrocyte activation, proliferation, and inflammasome activation, providing a reliable theoretical basis for afatinib in the treatment of neuroinflammation (39).

#### 4.1.2 New Indications

Activation of EGFR was found in neurodegenerative diseases, and afatinib was found to be used in the treatment of neuroinflammatory (39).

#### 4.1.3 Novel Combination Regimens

Combination of afatinib and temozolomide can cooperatively inhibit the proliferation, clonal survival, motor, invasive, and induced aging of glioblastoma (GBM) (43). Also, afatinib combined with paclitaxel and bevacizumab have a good antitumor activity, and the incidence of adverse reactions is under control (44). The favorable antitumor activity for afatinib with carboplatin and triple therapy of afatinib with carboplatin and paclitaxel are observed (45). In addition, head and neck squamous cell carcinoma (HNSCC) and the human papillomavirus (HPV) therapy with HER receptors, anti-EGFR therapies have shown that (46) afatinib monotherapy or afatinib combined with carboplatin can increase the sensitivity of cetuximab‐resistant cells. Beyond that, inhibition of autophagy may address the TKI resisitance of EGFR-mutant advanced NSCLC (38, 39).

### 4.2 Ibrutinib

#### 4.2.1 New Mechanisms

Although ibrutinib is an irreversible covalent inhibition of BTK, it showed good therapeutic effect and safety in the treatment of the first-line and relapsed/refractory CLL/SLL. In the meantime, the patients without chromosome 17p deletion benefits most from ibrutinib on the progression-free survival rate and overall survival. However, the long-term effects of ibrutinib on immune function of users still need further investigation (47, 48).

The sensitivity of ibrutinib treatment is related to unmutated igvh status and elevated zap70 expression and trisomy 12. Meanwhile, del17p/TP53 mutation is the inherent drug resistance factor of ibrutinib cells, which is not affected by the acquired BTK and PLCγ mutations and directly related to ibrutinib resistance (49). Arq531, a reversible BTK inhibitor, can inhibit BTK function, including B-cell receptor (BCR) signaling, activity, migration, CD40 and CD86 expression, and NF-κB gene transcription *in vitro*. Arq531 can obtain better survival rate than ibrutinib, and BCR-mediated C481S-BTK and PLCγ2 mutations, which are directly related to ibrutinib resistance, are also inhibited by Arq531 (50). In addition, the birth of daratummab makes CD38 a research hotspot. Daratummab has a good therapeutic effect on CLL through ADCC, CDC, and ADCP, and apoptosis mechanisms CD38 reduced the enhancement effect of ibrutinib on Syk, BTK, PLCγ2, ERK1/2, and AKT. Combined medication can target BTK and CD38 at the same time and exert a strong anti-CLL effect (51).

The HDAC inhibitor combined with ibrutinib caused a significant inhibition of p-IRE1 and p-BTK, thus inhibiting the downstream target of BCR, which is the effect that drug alone does not have (52). Furthermore, inhibiting BTK and P13K/mTOR simultaneously can enhance the therapeutic effect of ibrutinib on PCNSL with CD79B mutation (53). In the meantime, ibrutinib can also reduce the drug resistance of tumor cells to paclitaxel by inhibiting the outflow function of the ATP-binding cassette subfamily B member 1 (ABC B1/P-glycoprotein) and subfamily C member 10 (ABCC 10/MRP 7) (54). Also, ibrutinib inactivates EGFR in hepatocellular carcinoma cells and block downstream Akt and ERK signals, thus inhibiting the expression of key genes involved in cell proliferation, migration, and survival and activating cell differentiation (55).

#### 4.2.2 New Indications

Ibrutinib has an activity in relapsed or refractory primary central nervous system lymphoma (PCNSL), which shows resistance in PCNSL treatment (53). Ibrutinib has a good therapeutic activity on hepatocellular carcinoma, including sorafenib-resistant HCC cells (55).

#### 4.2.3 Novel Combination Regimens

Daratummab with ibrutinib have achieved the cancer cell killing effect *in vivo* and *in vitro* (51). Also, Arq531 has the potential to become the combination candidate drug of ibrutinib to overcome the resistance to ibrutinib (50). In addition, the participation of ibrutinib can promote the antitumor activity of paclitaxel (54).

Pan-class I/II histone deacetylase (HDAC) inhibitors can be used to treat some lymphomas, and ACY-1215 is the first selective inhibitor of HDAC. ACY-1215 plus ibrutinib is highly synergistic in lymphoma cell lines and primary human lymphoma samples. In the xenograft lymphoma model, this combination caused the tumor growth delay and prolonged the overall survival rate (52). Ibrutinib has synergistic effect with sorafenib or sorafenib homolog on apoptosis of hepatocellular carcinoma cells. *In vivo* test, the combination drug shows satisfactory effect and safety, and BTK + immune cells are enriched in tumor microenvironment (55).

## 5 The Chiral Small Molecular Targeted Antitumor Drugs Approved by the FDA in 2014

Idelalisib (6) was approved by the FDA to its list for recurrent follicular cell non-Hodgkin lymphoma (FL) and recurrent small lymphocyte lymphoma, in conjunction with rituximab for the treatment of recurrent chronic lymphocytic leukemia. (S)-Idelalisib is the active enantiomer.

### 5.1 Idelalisib

#### 5.1.1 New Mechanisms

High-frequency CMV reactivation and pneumocystis jiroveci pneumonia (PJP) were observed in the treatment of CLL using idelalisib. Mechanically, idelalisib impaired T-cell-mediated CMV responses and patients with gastrointestinal reactions were found to increase the percentage of Tregs in biopsy, sometimes associated with a positive PCR of infectious pathogens (56). Therefore, it can be speculated that idelalisib may have a side effect of T-cell damage that promotes infection or viral reactivation. Also, idelalisib causes impaired polymorphonuclear neutrophil (PMN) function, which in turn causes neutropenia-like susceptibility to infections (57).

Idelalisib could enhance the bendamustine-mediated DNA damage/repair response. In the meantime, γH2AX was separately activated and the corresponding translation process was synergfacilitated by the two drugs. A decrease in the MCL-1 total protein population in CLL cells was observed, and MCL-1-deficient heterogeneous mouse embryonic fibroblasts are highly sensitive to monotherapy and combined therapy (58).

In addition, idelalisib may increase the side effects of radiotherapy. One patient developed strong grade 2 radiodermatitis and grade 3 mucositis after 20 Gy radiotherapy, and idelalisib patients who do not take idelalisib show good tolerance, with the most serious adverse reaction being no more than grade 1 radiodermatitis (59). When the irradiation intensity was 2 Gy and idelalisib was 100 nmol/L, the radiosensitivity increased significantly, which may be directly related to the inhibitory effect of idelalisib on PI3K.

Idelalisib promotes Bim induction *via* the FoxO3a pathway after PI3K/AKT inactivation to induce apoptosis. This shows that Bim plays an important role in the treatment of HCC by idelalisib (60). Idelalisib induced PUMA *via* the AKT/GSK-3β/NF-κB pathway, p53 upregulated modolator of apoptosis, belonging to BH3-only Bcl-2 family, which play a key role in apoptosis in cancer cells (59). Interestingly, idelalisib can inhibit platelet aggregation mediated by ITAM receptors GPVI and CLEC-2 as well as the adhesion and was antithrombotic and bleeding at high doses (61).

#### 5.1.2 New Indications

Idelalisib showed good activity in HCC cells and colon cancer cells (59, 60). In addition to this, idelalisib can collaborate with 5-FU or regorafenib to induce colon cancer apoptosis, with PUMA involvement in the process, which can serve as the sensitivity index of idelalisib in the treatment of colon cancer, and it is also the main factor of the role of idelalisib of anticolon cancer (61).

#### 5.1.3 Novel Combination Regimens

Idelalisib + bendamustine + rituximab therapy showed greater efficacy and safety compared with bendamustine and rituximab therapy commonly used clinically to treat recurrent/refractory CLL (62). Bindem therapy by idelalisib + bendamustine produced synergistic cytotoxicity in CLL therapy (58). However, the safety evaluation of idelalisib, denidamide, and rituximab showed excessive toxicity, thus the combination is recommended in a carefully designed and diligently tested clinical trial environment (63). The results of the effect of idelalisib on the treatment of rituximab and obinutuzumab on leukemia showed that inhibition with idelalisib on PI3K did not negatively affect the efficacy of the above two McAb and had clinical value associated with them (64). Meanwhile, idelalisib and sorafenib or doxorubicin exhibit synergistic anti-HCC effects, in which the decrease of anti-HCC effect of idelalisib in Bim-deficient objects is observed (60).

## 6 The Chiral Small Molecular Targeted Antitumor Drugs Approved by the FDA in 2015

There were three drugs approved for listing by the FDA, including ixazomib (7), sonidegib (8), and cobimetinib (9). Ixazomib combination with lenalidomide and dexamethasone are approved for patients with multiple myeloma who have been treated at least one time. Sonidegib is approved for local advanced basal cell cancer that has recurred after or is not suitable for surgery or radiotherapy. Cobimetinib is approved in association with vemurafenib (or vemurafenib +atezolizumab) for melanoma with BRAF V600E or V600K mutations.

### 6.1 Ixazomib

#### 6.1.1 New Mechanisms

Ixazomib enhances the activation of the PTH-induced β-catenin/TCF signal by inducing PTHR separation from β-catenin, thus enabling the regulation of the PTHR signal to maintain the PTH anabolic effect (65). In HCT116 p53, ixazomib is induced by CHOP-dependent DR5, sensitizing the tumor apoptosis process induced by tumor necrosis factor-related apoptosis-inducing ligand (TRAIL) (66).

#### 6.1.2 New Indications

In addition to treat MM approved by the FDA, ixazomib also improves the treatment effect of PTH on osteoporosis and other absorptive bone diseases (65). In the treatment of solid tumors, ixazomib showed therapeutic activity in the treatment of colorectal cancer (CRC) and triple-negative breast cancer (TNBC) (66).

#### 6.1.3 Novel Combination Regimens

In the treatment of recurrent/refractory acute myeloid leukemia, the maximum tolerated dose of ixazomib with mitoxantrone, etoposide, and cytarabine (MEC) was 1.0 mg, observing the dose-dependent thrombocytopenia as the controlled toxicity, and the overall response rate reached 53%, indicating that the combination drug had certain therapeutic effect (67).

The China Continuation study results support the extension of ixazomib + lenalidomide + dexamethasone (IRD) therapy in RRMM therapy worldwide (68). Also, there was also a significant association between ixazomib exposure and the adverse reactions of triple therapy, the probability of not only anemia and thrombocytopenia above level 3 but also diarrhea fatigue, nausea, peripheral neuropathy, and rash were directly related with Ixazomib exposure, which can be controlled by adjusting the dose of ixazomib, providing a reference to the clinical dose range of ixazomib (69).

In NDMM therapy, two triple therapies for Ixazomib were proposed. One is the ixazomib + RD (IRD), which shows good therapeutic efficacy and safety in patients who have not received autologous stem cell transplantation (SCT). Subsequent maintenance treatment can be treated with ixazomib alone (70). The other is the ixazomib-melphalan-prednisone (IMP) which showed good tolerance and antimyeloma activity. A single dose of ixazomib can be used for maintenance treatment in elderly NDMM patients and those who are not eligible for transplantation (71). In addition, IDR showed good therapeutic activity and safety in Waldenstrom macroglobulinemia (WM), providing a safe, simple, and effective treatment for patients with WM (72).

*In vivo* metabolism of ixazomib provides new ideas for its drug combination. When the concentration of ixazomib exceeds the clinical level, ixazomib was observed to be metabolically metabolized by multiple CYP isoenzymes. The effect of CYP3A inhibitors on ixazomib metabolism was not significant, which could be used directly together without adjusting to the dose of ixazomib. For strong CYP3A inducers, simultaneous administration with ixazomib should be avoided, as which significantly reduces the whole body exposure of ixazomib, somewhat reducing the efficacy of ixazomib (73). Furthermore, ixazomib with carboplatin had good treatment for TNBC and patients demonstrated good tolerance to bindem therapy in the second stage of the trial (74).

### 6.2 Sonidegib

#### 6.2.1 New Mechanisms

Debilitating taste disorder was reported in patients using hedgehog pathway inhibitior (HPI). The mouse had a loss of taste after using sonidegib, but the response of the tongue did not change to tactile stimulation. Rats and mice had the same performance in neural effects in fungiform (FP), and rats were significantly more severe than mice in taste buds (TB) and circumvallate papillae (CV) (75). For patients with high-risk limited prostate states undergoing radical prostatectomy, GLI1 expression identified baseline levels of hedgehog signaling pathway activity after the use of sonidegib. Sonidegib can reach the prostate site and produce a 60-fold hedgehog inhibition effect (76). In the human and mouse chronic graft-versus-host disease (cGVHD), the hedgehog signal is active, accumulating the transcription factors GLI-1 and GLI-2 particularly in fibroblasts, leading to the pathologic fibrosis seen in cGVHD. However, H-score for sonic hedgehog (Shh), theoretically not affected by sonidegib, showed a significant content decline *in vitro*, for unclear reasons (77).

#### 6.2.2 New Indications

Sonidegib suppresses the hedgehog signaling pathway and is thought to be used in the adjuvant treatment of prostate cancer. However, the significance and status of sonidegib in prostate cancer have not been clarified (76). Sonidegib has the potential to treat steroid refractory cGVHD, but the patient showed obvious toxicity accumulation so as to discontinue the treatment. The sonidegib adverse reactions and the pathological characteristics of cGVHD partially overlap, and it was difficult to distinguish the specific correlation between toxicity and sonidegib. This partly limits the use of sonidegib in cGVHD (77).

In addition, sonidegib has good efficacy and safety for locally advanced basal cell carcinoma. Moreover, positive results from trials conducted in patients with Gorlin syndrome showed the possibility of using sonidegib not only for treatment of locally advanced basal cell carcinoma but also for cancer chemoprevention (78).

#### 6.2.3 Novel Combination Regimens

The combination of sonidegib and ruxolitinib can produce good tolerance and safety in patients with multiple myeloma who have not used JAK inhibitors. The study identified a combined recommended phase 2 dose (RP2D) regimen for sonidegib 400 mg daily + ruxolitinib 20 mg twice daily. The overall benefits of combined therapy compared with ruxolitinib alone are relatively limited and do not have significant advantages for the treatment effect of multiple myeloma, which is a near step to extended clinical trials of sonidegib (79).

### 6.3 Cobimetinib

#### 6.3.1 New Mechanisms

Using cobimetinib combined with vemurafenib for the treatment of melanola, the the MAPK signaling pathway activated by MEK is the core cause of antagonizing BRAF inhibitors (80). There is no toxicity accumulation in long-term treatment, but it was reported that (81) cobimetinib caused blurred vision and eye photophobia in the treatment of metastatic melanoma with BRAF mutations, and the symptoms were basically resolved 14 days after treatment. The specific mechanism is still unclear. In addition, cobimetinib developed very rare “dropped head syndrome” in treating patients with Erdheim‐Chester Disease (ECD), which were relieved by reducing the administration dose after a period of withdrawal (82).

BCL2 protein was enriched in leukemia progenitors and that cobimetinib inhibited cytokine-induced pERK and PS6 signaling pathways. The signaling pathway downstream of MAPK is inhibited and is synergistic in apoptosis of cancer cells. Cobimetinib combined with venetoclax downgrades the content of the MCL1 protein, and the BCL2:BIM and MCL1:BIM complexes are damaged and release BIM, thus causing apoptosis of cancer cells (83). The BRAF-mediated MEK/ERK-mediated MCL-1 upregulation is the production mechanism of colorectal cancer cell resistance that causes BRAF mutations. Cell experiments found that combination of cobimetinib and MCL-1 antagonists significantly inhibited growth of tumor cell growth and antagonized cell resistance (84).

Higher expression of FLT3 and MDM2 in AML cells of normal karyotype (NK) and wild-type TP53, and are therefore most sensitive to combination therapy. As a result, the content of FLT3 and MDM2 can be used as biomarkers for the combination therapy of cobimetinib and idasanutlin for AML (85).

#### 6.3.2 Novel Combination Regimens

The drug combination of dabrafenib and trametinib was shown to have a therapeutic effect comparable with the drug combination of cobimetinib and vemurafenib, and the former was safer. However, the study only provided data reference for doctors (86). Cobimetinib combined with venetoclax can play the therapeutic activity against leukemia, targeting both BCL2 and MAPK pathways to induce apoptosis of cancer cells (84). In addition, cobimetinib combined with MDM2 antagonists idasanutlin showed significantly induced apoptosis in the AML cell line (85).

Duligotuzumab is a humanized monoclonal bispecific antibody that targets to inhibit HER3 and EGFR and inhibit downstream signaling of AKT and ERK. The combination of duligotuzumab and cobimetinib in the treatment of KRAS-mutated tumors showed poor resistance and limited efficacy in the subject population. However, cobimetinib and duligotuzumab as single combined with other drugs achieve better tolerance (86).

The accumulation and survival of tumor-specific T cells is facilitated by MEK inhibitors. In the treatment of solid tumor, the cobimetinib and PD-L1 inhibitors atezolizumab has good safety and therapeutic activity and is unaffected by KRAS/BRAF activity (83, 87). In mouse models of colorectal cancer with BRAF V600E mutations, cobimetinib combined with MCL-1 antagonists showed good drug-resistant antagonism (88).

## 7 The Chiral Small Molecular Targeted Antitumor Drugs Approved by the FDA in 2017

Niraparib (10), acalabrutinib (11), and midostaurin (12) were approved to its list by the FDA in 2017, niraparib for patients with recurrent ovarian, tubal, or primary peritoneal cancer completely or partially relieved after platinum chemotherapy. Acalabrutinib is for patients with CLL or SLL. Midostaurin is for newly diagnosed *FLT3* gene mutations (combined with chemotherapy drugs) and adult aggressive systemic hypergalocytosis (ASM), systemic hypergalocytosis (SM-AHN) with blood tumors, and hypertrophic cell leukaemia (MCL). Both (S)-niraparib and (S)-acalabrutinib are active enantiomers. The active enantiomer of midostaurin is (5S, 6R, 7R, 9R)-enantiomer.

### 7.1 Niraparib

#### 7.1.1 New Mechanisms

Niraparib was metabolized by hydrolysis and binding pathway, 31.6% of drugs from feces and 40.0% of drugs from urine 14 h after administration. About 29.9% of the fecal and urine excretion drugs are drug prototypes. According to research data, niraparib is a low liver extraction drug with high bioavailability, low clearance, and long half-life, in line with the anticancer activity of niraparib (89, 90). Improved time without symptoms or toxicity has been demonstrated in the niraparib-treated patients of recurrent ovarian cancer (91).

Niraparib has been shown to regulate the tumor immune microenvironment. In breast cancer cell lines and xeno-transplantation models, PARP inhibitors upregulate PD-L1 expression in a tumor inherent manner, whether BCRA is mutated or not. Mechanically, niraparib can enhance the activity of the the type I (alpha) and type II (gamma) interferon pathway and increase the infiltration of CD8 + and CD4 + cells in the tumor. Meanwhile, niraparib treatment alone may cause immune memory (92). In conclusion, good cancer effects may be because niraparib increased sensitivity of the tumor to immune checkpoint blocking therapy.

TWIST may be the oncogene that promotes OC cells to cisplatin resistance. In TWIST-deficient cisplatin-resistant OC cells (CisR OC), niraparib and cisplatin have synthetic lethal effects on cancer cells. Further research reveals two potential mechanisms, one by blocking the DNA repair, which suppresses the activation of PARP1 and XRCC1. The other is mitochondrial emergency-mediated apoptosis. Cytochrome *c* in the mitochondria is released into the cytoplasm, initiating cypase-dependent apoptosis, resulting in irreversible cell death (93).

When the niraparib dose increased to 300 mg, apalutamide led to a higher incidence of dose-limiting toxicities (DLTs). In contrast, AAP and niraparib have better tolerance and security. The researchers speculated that apalutamide induced the metabolism of niraparib, reducing niraparib exposure or that apalutamide induced the P-glycoprotein to reduce niraparib exposure. Therefore, the efficacy of apalutamide combined with niraparib is affected (90).

#### 7.1.2 New Indications

Niraparib could prolong the survival of patients with platinum-sensitive recurrent ovarian cancer who responded to the last platinum chemotherapy. Furthermore, maintenance therapy with niraparib benefits regardless of the patient’s response to the last platinum chemotherapy (94).

#### 7.1.3 Novel Combination Regimens

Niraparib combined with PD-1 inhibitors showed good tolerance and controllable safety in the treatment of recidivity ovarian cancer (95). Meanwhile, niraparib and pemburolizumab achieved 21% objective remission and 49% in advanced or metastatic TNBC patients (96). The safety of combined treatment is controlled and has further clinical research.

Niraparib combined with three commonly used anti-CRC drugs (5-fluorouracil, oxaliplatin, or irinotecan) and the therapy of niraparib and irinotecan were the strongest (97). When niraparib and apalutamide or abiraterone acetate plus prednisone (AAP) were used to treat metastatic castration-resistant prostate cancer (mCRPC), two different combination therapies showed safety differences. Apalutamide may cause patient fatigue (90).

### 7.2 Acalabrutinib

#### 7.2.1 New Mechanisms

In human CLL NSG heterhorygraft models, acalabrutinib reduces phosphorylation of PLCγ2 and ERK and significantly inhibit CLL tumor cell proliferation. In TCL1 overrelay transfer models, acalabrutinib can inhibit phosphorylation of BTK, PLCγ2, and S6. At concentrations below 10 nm, ibrutinib produces varying levels of inhibition on all nine kinases containing cysteine residues, while acalabrutinib inhibition only on BTK. Acalabrutinib showed high selectivity and showed similar treatment effects to ibrutinib (98). Twice daily alone of acalabrutinib in recurrent/refractory cell lymphocyte lymphoma achieved a high percentage of overall and complete efficacy (99, 100).

#### 7.2.2 New Indications

Acalabrutinib shows good safety and tolerance in patients with CLL or SLL who stop taking medication with adverse reactions using ibrutinib (101).

#### 7.2.3 Novel Combination Regimens

In recurrent/refractory cell lymphocyte lymphomathe, the combination of acalabrutinib and obinutuzumab can further optimize the security of acalabrutinib and specifically improve the adverse events inherent in acalabrutinib (100).

The potential drug association protocol was identified by analyzing the pharmacological characteristics of CLL patients treated with acalabrutinib, in the BCL-2 inhibitor venetoclax, alkylazer bendamustine, proteassomal inhibitor carfilzomib, and nucleoside analog fludarabine, duvelisib (PI3K inhibitor). In ACP-319 (in PI3K delta inhibitor), both *in vivo* and *in vitro* experiments show that venetoclax can maximize acalabrutinib to play anticancer effect, and both the degree and safety of apoptosis of cancer cells were optimized (102).

Meanwhile, anti-CD20 antibodies combined with highly selective BTK inhibitors can achieve better anti-CLL effects. Obinutuzumab and acalabrutinib for CLL found that (103) 95% of patients previously ineffective and 92% of patients with refractory or relapse, indicating that combined therapy could achieve a high level of treatment effect, but 71% of patients had grade 3/4 adverse reactions and safety requires further research. In addition, acalabrutinib monotherapy and acalabrutinib combined with obinutuzumab all showed superior treatment effects than obinutuzumab-chlorambucil (104).

Acalabrutinib combined standard care CHOP-R chemical immunotherapy (cyclophosphamide, doxorubicin, vincristine, prednisolone, and rituximab) in a new diagnosis of DLCBL type Richter’s syndrome (RS) for CLL transformation (105). Clinical treatment of CD19 + B cells usually used chimeric antigen receptor (CAR) T-cell therapy. Lisocabtagene maraleucel (liso-cel) is a drug candidate for recurrent/refractory non-Hodgkin lymphoma or CLL. Acalabrutinib combined with CAR-T cells can increase the mortality of CD19 + tumor cells and prolong the survival of charged tumor mice. It can be preliminarily argued that the combination of liso-cel and acalabrutinib can enhance the therapeutic effect of CAR-T-cell therapy that has been found in CD19 + B-cell malignancies (106).

### 7.3 Midostaurin

#### 7.3.1 New Mechanisms

Midostaurin can induce MCL-1 downregulation, thus enhancing the activity of venetoclax, which inhibited BCL-2, in turn leading to Bim release, causing apoptosis. The presence of continuously active and enhanced spleen tyrosine kinase (SYK) in FLT3-ITD-positive AML, its overexpression affects the transformation of AML and its resistance to FLLT3 inhibitors (107). Also, midostaurin had excellent inhibitory effects on FLT3-ITD cells and was 100 times more potent on FLT3-ITD or FLT3-ITD + TEL-SYK cells than SYK inhibitors. The main driver of SYK-induced cell conversion was STAT5, and midostaurin combined with FLT3/SYK dual inhibitor or SYK alone both have good inhibition of STAT5 (108–110).

Multidrug resistance (MDR) is a common cause of chemotherapy failure, and the overexpression of ABC transporters is probably the cause. Midostaurin has been proved to antagonize ABCB1-mediated MDR, but it cannot reverse ATP-binding cassette subfamily G member 2 (abcg2)-mediated MDR. Also, midostaurin directly inhibited the efflux function of ABCB1 transporter and inhibited the ATPase activity of ABCB1 transporter in a dose-dependent manner. Midostaurin combined with chemotherapy may improve the therapeutic effect of tumor (111).

In the treatment of NSCC, midostaurin inhibits TBK1, PDPK1, and AURKA at the same time, and the combined inhibition of these targets changed PI3K/AKT and cell cycle signaling pathways, which were partially concentrated on PLK1 (112).

#### 7.3.2 New Indications

Acquired KIT D816V mutation widely exists in patients with advanced systemic mastocytosis (advSM) and also exists in mast cells and other hematopoietic cell lines. Midostaurin can target mast cell chamber and KIT-D816V-positive AHN but may not be able to overcome high molecular risk mutation (S/A/R gene panel). Compared with midostaurin, avapritinib, another KIT inhibitor, showed better *in vitro* activity even when midostaurin did not respond (113). In additin, midostaurin showed good activity in NSCC treatment (114).

#### 7.3.3 Novel Combination Regimens

Adding midostaurin to standard chemotherapy in AML patients with FLT-3 mutations can significantly prolong the total and event-free survival and improve patient prognosis. Midostaurin combined with BCL-2 inhibitor venetoclax has a good therapeutic effect on FLT3-ITD AML (107).

For kinase inhibitor-sensitive/resistant diseases, SYK and FLT3/SYK dual inhibitors can increase midostaurin growth inhibition on cancer cells, providing a reliable reference for the clinical combination regimen of midostaurin. FLT3-ITD-positive AML with a high allele ratio (>0.5) had a poor prognosis; midostaurin combined with the MDM2 inhibitor NVP-HDM201 provides significant therapeutic effects on the wild-type AML of high allelic FLT3-ITD ratio by targeting P53 and NPM1 (110). Also, for AML without FLT3 mutation, midostaurin showed synergistic inhibition with standard chemotherapy drugs and some targeted drugs (112). At the same time, the combination of midostaurin and PLK1 inhibitor was observed to have significant synergistic inhibitory effect on lung cancer cells (114).

## 8 The Chiral Small Molecular Targeted Antitumor Drugs Approved by the FDA in 2018

There are seven chiral small molecular targeted antitumor drugs approved to its list by the FDA in 2017. Encorafenib (13) is for unresectable or metastatic melanoma patients with BRAF V600E or BRAF V600K mutation and treat metastatic colorectal cancer (mCRC) patients with BRAF V600E mutation (combined with cetuximab). Ivosidenib (14) is for recurrent or refractory AML in human with IDH1 mutation and AML patients aged 75 and over who could not use intensive chemotherapy due to other complications. Duvelisib (15) is for recurrent or refractory chronic lymphocytic leukemia(R/R CLL), small lymphocytic lymphoma (SLL), and recurrent or refractory follicular lymphoma. Talazoparib (16) is for locally advanced or metastatic breast cancer with BRCA mutation (harmful or suspected harmful) and HER2 negative. Lorlatinib (17) is for ALK-positive metastatic NSCLC. Larotrectinib (18) is for adult and child patients with locally advanced or metastatic solid tumors with *NTRK* gene fusion. Glasdegib (19) is for untreated AML (combined with low-dose cytarabine).

The active enantiomers of encorafenib, ivosidenib, duvelisib, and lorlatinib are (S)-enantiomers. (S,R)-Enantiomers of talazoparib and larotrectinib are approved to its list by the FDA. The active enantiomer of glasdegib is (R,R)-enantiomer.

### 8.1 Encorafenib

#### 8.1.1 New Mechanisms

The generation and activation of RAF dimer will lead to the reactivation of MEK-ERK pathway, which is the reason why colorectal cancer is resistant to BRAF inhibitors. PLX8394, a Paradox breaker BRAF inhibitor, can inhibit the formation of RAF dimer. PLX8394 and encorafenib have higher anticancer efficacy than vemurafenib, and the reactivation degree of MEK-ERK pathway is lower. The dose-response curves of PLX8394 and encorafenib are similar, but there is no significant difference in the reactivation degree of MEK-ERK pathway (115, 116).

#### 8.1.2 Novel Combination Regimens

The FDA approved encorafenib combined with cetuximab to treat mCRC with BRAF V600E mutation. The activation of PI3K/AKT pathway is considered to be the mechanism of resistance of mCRC to BRAF inhibitors. Generally speaking, the addition of PI3K inhibitor may indeed improve the prognosis of mCRC patients treated with encorafenib combined with cetuximab, although the incidence of adverse events is higher (117).

The triple regimen of Encorafenib combined with cetuximab plus MEK inhibitor binimetinib showed good tolerance and safety in the treatment of mutant mCRC (115). The therapeutic effects of dabrafenib/trametinib, vemurafenib/cobimetinib, and encorafenib/binimetinib on BARF mutant mCRC were compared in parallel. The encorafenib/binimetinib joint scheme has the longest OS time and higher security. The adverse event of dabrafenib/trametinib regimen is fever, and that of vemurafenib/cobimetinib regimen is photosensitive reaction. Therefore, encorafenib combined with binimetinib has superior curative effect and tolerance (118). Clinical trials of encorafenib and binimetinib combined immunotherapy for melanoma are in progress, for example, two combined clinical trials of CTLA4 antibody ipilimumab and PD1 antibody pembrolizumab (119).

A total of 60% of patients with BRAF mutant MBMs had brain metastasis, which seriously affected the treatment effect and prognosis of patients. The treatment of encorafenib combined with binimetinib shows the effect of resisting melanoma brain metastasis, and the specific efficacy and safety need further study. In addition, metastatic melanoma is easily resistant to BRAF inhibitors, and serine synthesis may be the cause of drug resistance (120). Furthermore, antifolate methotrexate can be used as sensitizer for BRAF inhibitors dabrafenib and encorafenib. At the same time, the activation mutation of RAS codon 12 is a prognostic marker of the therapeutic effect of methotrexate combined with BRAF inhibitor (121).

### 8.2 Ivosidenib

#### 8.2.1 New Mechanisms

The mutation of IDH1 and IDH2 can cause excessive production of D-2-hydroxyglutaric acid (2-HG) and impaired cell differentiation. RTK pathway mutation and 2-HG restoration mutation (including isomer transformation and the appearance of mIDH1-S280F) are the mechanisms of ivosidenib secondary drug resistance. Furthermore, 2-HG can enter specific cell types, such as tumor-associated immune cells, which may lead to immunosuppression. The emergence of multiple nondominant mutations at the second site of IDH1 can be used as a basis for the combination of mIDH1 inhibitors and other therapies, which will reduce the probability of ivosidenib resistance (122).

#### 8.2.2 New Indications

In the treatment of mIDH1 cholangiocarcinoma without progression after chemotherapy, ivosidenib can significantly prolong the progression-free survival (PFS), prolong OS to 10.8 months, and reduce the risk of disease progression and death by 63%, which provides a reliable basis for ivosidenib to treat mIDH1 cholangiocarcinoma. Ivosidenib is suitable for the treatment of advanced mIDH1 cholangiocarcinoma (123).

Ivosidenib has a certain potential in the treatment of advanced mIDH1 chondrosarcoma, which was well tolerated, had no dose-limiting toxicity, and almost had no treatment-related adverse events of grade ≥3. Biopsy report showed that the content of 2-HG decreased significantly after ivosidenib was used. Ivosidenib has the potential to be a candidate drug for patients with advanced mIDH1 chondrosarcoma without treatment options (124).

#### 8.2.3 Novel Combination Regimens

For mIDH1 AML patients with FLT3 and RAS mutations, dual treatment with kinase inhibitors has certain therapeutic potential. The dual pharmacological inhibition of mIDH1 and mIDH2 may alleviate the restoration of 2-HG caused by homotypic transformation. In addition, the combination with nontargeted drugs can also improve the prognosis. Ivosidenib combined with intensive chemotherapy drug enasidenib can enhance induction and consolidate treatment for mIDH1/2 AML. Its safety and tolerance are also within the acceptable range (125).

In the treatment of solid tumors (cholangiocarcinoma, osteosarcoma, etc.), ivosidenib shows good oral exposure, rapid absorption, and long terminal half-life after single administration (average 40–102 h after single administration). The accumulation of ivosidenib in tumor reached a stable state after 15 days of administration, which was moderate accumulation. The reduction of 2-HG reached 98%, which has reached the health standard. The disease characteristics of patients and the concurrent administration of weak CYP3A4 inhibitor/inducer did not affect the exposure of ivosidenib. Ivosidenib at 500 mg q.d. is an appropriate dose (126). Ivosidenib can be used to treat mIDH1 glioma which showed brain penetrance and decreased 2-HG compared with the control group (127).

### 8.3 Duvelisib

#### 8.3.1 New Mechanisms

In the combination of duvelisib with ibrutinib or dexamethasone, the inhibition of phosphorylated (p) Akt at serine473 was observed within 12 h after duvelisib application, and the reactivation of mTOCR2-dependent pAKT was obvious within 24 h. Combined medication significantly inhibited the activation, prolonged the inhibition time of pAKT, and further inhibited the survival and growth signal mediated by mTORC1/2 (128).

Mechanically, duvelisib inhibits PI3K-δ/γ, and then upregulates the apoptosis-promoting BH3-only protein, thus initiating the apoptosis process of CLL cells. Cell survival is caused by the upregulation of BCL-2. Venetoclax can inhibit BCL-2, thus increasing the degree of apoptosis. At the same time, Duvelisib can reduce MCL-1 mRNA and expressed protein to a small extent, because MCL-1 can promote the survival of malignant lymphoma cells, which may increase the sensitivity of CLL cells to venetoclax[134.

#### 8.3.2 New Indications

Duvelisib is superior to ofatumumab in efficacy and safety in the treatment of R/R CLL/SLL and can improve the PFS of patients (129).

#### 8.3.3 Novel Combination Regimens

Through high-throughput collaborative screening (128), it was found that the combination of duvelisib and several drugs has significant activity *in vivo* and *in vitro*, especially in the treatment of duvelisib combined with ibrutinib or dexamethasone.

The treatment of duvelisib combined with venetoclax can cause more apoptosis of CLL (130). An Ib/II study discussed the efficacy and safety of duvelisib combined with fludarabine, cyclophosphamide, and rituximab (FCR) in the treatment of young patients with CLL. The second-stage dose was determined to be 25 mg b.i.d. About 2/3 of patients reached BM-uMRD. However, 73% of patients’ 3-year PFS did not show significant advantages compared with FCR triple therapy. At the same time, duvelisib combined with FCR can cause common immune-mediated toxicity and infection complications, which can be controlled by intervention (131).

### 8.4 Talazoparib

#### 8.4.1 New Mechanisms

Temozolomide resistance often occurs in patients with glioblastoma. The mechanism of DNA repair is considered one of the possible reasons for poor temozolomide resistance. Mechanically, talazoparib enhances temozolomide by inhibiting BER-mediated repair of N3MeA and N7MeG DNA damage. PARP trapping produces protein-DNA complex in BER intermediate, which causes cytotoxicity and leads to apoptosis. The results of cell experiments *in vitro* further confirm this theory. Furthermore, talazorib had a significant tendency of multidrug resistance protein 1 (MDR 1) efflux, which probably caused talazorib to pass through the blood-brain barrier only in a small amount (132). This provides an explanation for the loss of TMZ sensitization mediated by talazoparib in GBM xenograft model.

There will also be resistance to radiotherapy in GBM treatment, which is directly related to the powerful DNA repair ability of glioblastoma stem cells (GSCs). Compared with photon irradiation combined with TMZ, talazoparib significantly reduced the active number of CSC in GBM cell lines and can obviously prolong G2/M phase tissues and inhibit cell proliferation (133).

A new functional IncRNA was discovered, which was named POLO oxykinase 4-related IncRNA (IncRNA PLK4). In the tissues and cells of hepatocellular carcinoma (HCC), the content of IncRNA RLK4 was significantly downregulated, while talazoparib could increase its expression. Furthermore, talazoparib could enhance the inactivation of YES-related protein (YAP) and cell aging by upregulating the content of IncRNA PLK4, thus achieving the purpose of inhibiting the survival and growth of hepatoma cells (134).

#### 8.4.2 New Indications

In the treatment of solid tumors, there was no clinical-related change in PR, QRS, QTcF, or RR interval, heart rate, or ECG morphology after treatment with talazoparib at 1 mg daily (135). Talazoparib has the potential to be an adjuvant therapy for breast cancer patients with gBRCA positive before operation. The patients took talazoparib orally every day before operation for 6 months without taking other chemotherapy methods, and the proportion of patients with RCB-0 (complete pathological remission) increased significantly, and the safety and tolerance were within the controllable range (136).

*In vitro* studies showed that talazoparib is the substrate of P-glycoprotein and breast cancer drug-resistant protein transporter. Itraconazole (P-glycoprotein inhibitor) increased the plasma exposure of talazobarib, and when P-glycoprotein inhibitor must be combined, the dosage of talazobarib should be appropriately reduced (from 1 mg to 0.75 mg/day). Rifampicin also caused a similar increase in exposure to talazoparib, which indicated that P-glycoprotein inducer had limited effect on talazoparib (137). In the meantime, the treatment of talazoparib combined with these two drugs has good tolerance and safety.

#### 8.4.3 Novel Combination Regimens

Talazoparib that inhibits DNA repair have the potential to become a means to solve temozolomide drug resistance (132). In addition to GBM radiotherapy, talazoparib can increase the sensitivity of SCLC to radiotherapy, which can effectively inhibit the sensitivity of SCLC cell line and xenograft model to radiotherapy, and high PARP trapping activity can improve the sensitivity of SCLC to radiotherapy (136). Also, talazoparib can make melanoma cells sensitive to radiotherapy, while healthy tissue cells are less affected, indicating that combined therapy has certain selectivity. Because the research shows great heterogeneity, it is best to detect tumor cells before using this therapy (138).

The combination of PARP inhibitor and APE1 inhibitor may be a candidate treatment for malignant hematological tumors, such as myelodysplastic syndromes/chronic myelomonocytic leukemia and acute myeloid leukemia. Also, talazoparib and APE inhibitor III shows remarkable antileukemia effect, meanwhile, low dose of talazoparib and APE inhibitor can enhance the cytotoxicity of decitabine inmyelodysplastic syndromes/chronic myelomonocytic leukemia and acute myeloid leukemia (139).

### 8.5 Lorlatinib

#### 8.5.1 New Mechanisms

Mechanically, both lorlatinib and crizotinib can inhibit endothelial cells, but lorlatinib is more obvious for hcmec/D3 (normalized human brain microvascular endothelial cells). In SH-SY5Y (human neurobionoma cells) hypoxia model, lorlatinib also showed better protection against injured nerve cells than crizotinib. In addition, lorlatinib can downregulate the expression of SPP1, VEGF, TGF-β, and claudin in brain tissue and upregulate the expression of early growth transcription factor (Egr1), which may lead to the decrease of tight junctions between BBB cells, thus increasing the permeability of blood-brain barrier, which leads to higher brain exposure of lorlatinib. At the same time, the protective effect of lorlatinib on nerve cells and its characteristic of not affecting the quantity of P-glycoprotein may be the reason why the central nervous system is less resistant to it (140).

Patients of ALK rearrangement lung cancer shows drug resistance to lorlatinib; these mechanisms include epitheilial-mesogenic transition susceptible to combined ALK/Src inhibition, ALK compound mutations, and a novel bypass mechanism, mediated by Nf2 loss and outcome by mtor inhibition (141). Continuous use of ALK inhibitors in prophase treatment is likely to promote the occurrence of ALK-compliant mutations. Lorlatinib, the third-generation ALK inhibitor, might avoid refractory ALK mutation in early treatment and optimize clinical treatment effect. Also, the highly targeted inhibition of lorlatinib may cause ALK-independent drug resistance, and this drug resistance mechanism is difficult to overcome once established (142).

#### 8.5.2 New Indications

Lorlatinib has the potential to treat ALK/ROS-positive NSCLC-inhibiting drug-resistant mutation, which has a good therapeutic effect on patients who have received more than two TKI treatments and failed to be treated, and its safety is in a controllable range (141). A patient with ALK-positive central nervous system disease was successfully treated with lorlatinib for central nervous system metastasis (142). The patient had previously used a large dose of brigatinib.

Lorlatinib is often more effective for patients who fail to be treated well using the second-generation ALK-TKI, and it is very important to further study the plasma and tissue genotyping of ALK mutations and make a prospective prediction of ALK-TKI types for ALK resistance mutations of patients, which is very important for ALK TKI selection of advanced ALK-positive NSCLC patients (143, 144).

In the treatment of advanced ALK/ROS-positive NSCLC patients with drug-resistant mutations, lorlatinib, as a sequential drug in the treatment of brigatinib and alectinib, can significantly exert its therapeutic activity, and patients have good tolerance to lorlatinib without serious adverse reactions (145). Crizotinib and entrectinib have been approved for the treatment of advanced ROS1 rearrangement lung cancer, and lorlatinib has shown good activity in crizotinib-resistant environment. When the drug resistance of crizotinib and entrectinib is ROS1-G2032R mutation mediated by nontargeted drug resistance, lorlatinib can be used as the second-line treatment for ROS1-rearranged lung cancer, which can appropriately prolong the progression-free survival time of patients (146).

#### 8.5.3 Novel Combination Regimens

Compared with crizotinib, lorlatinib is intended for first-line treatment, and the best solution is to use lorlatinib as a combination drug to avoid ALK-dependent and ALK-independent drug resistance (147).

### 8.6 Larotrectinib

#### 8.6.1 New Mechanisms

The chimeric protein encoded by NTRK after rearrangement has carcinogenic effect, and at the same time drives constitutive expression and ligand-independent activation. Larotrectinib has a wide range of anti-NTRK fusion cancer effects, regardless of cancer type, age, and fusion partner (148). It is necessary to add NTRK status to the diagnosis workflow of tumor types, which means that some patients will benefit from targeted therapy.

#### 8.6.2 New Indications

Larotrectinib is used in children with locally advanced TRK fusion sarcoma, which can help the subsequent surgical resection of sarcoma, which is expected to be a preoperative drug for children with newly diagnosed TRK fusion sarcoma (149). When larotrectinib is used in infants, children, and adolescents, the recommended second-stage dose is 100 mg/m², which has nothing to do with the age of patients. Also, high response rate and good tolerance have been observed (150). Patients with advanced childhood cancer should be screened for TRK fusion, so as to determine whether larotrectinib can be used for preoperative treatment. Safety data confirmed the feasibility of long-term use of larotrectinib (151).

Rare patients with cervical sarcoma will become the beneficiary group of targeted therapy (152). Larotrectinib also shows certain therapeutic activity in the treatment of infant glioblastoma driven by NTRK, but there is a lack of long-term clinical research data of larotrectinib and this group. At the same time, small molecule targeted therapy may interact with radiotherapy. Therefore, the efficacy and safety of larotrectinib in the treatment of infant glioblastoma need to be further studied (153).

In addition, a clinical study of a Ph-like ALL case presents two genome mutations, one NRASGly12Asp mutation and one ETV6-NTRK3 rearrangement, which activate signal transduction. This case is resistant to many chemotherapies and immunotherapies and cannot avoid the recurrence after treatment (154). Researchers speculate that larotrectinib may be used in the early stage of the disease to achieve better curative effect.

### 8.7 Glasdegib

#### 8.7.1 New Indications

In the treatment of primary/secondary MF treated with at least one JAKI, glasdegib was safe and tolerant when used alone. Also, the study confirmed that glasdegib has a long-term sustained treatment response to MF (155).

#### 8.7.2 Novel Combination Regimens

The therapeutic effect and controllable safety of glasdegib combined with cytarabine have been proved on newly diagnosed AML patients (156). It is preliminarily determined that patients with moderate or severe renal damage may not need to reduce the initial dose of glasdegib (157).

Glasdegib combined with cytarabine showed better therapeutic effect than cytarabine alone. Glasdegib showed population-related adverse events, such as hematological events, gastrointestinal toxicity and fatigue, and adverse events related to hedgehog inhibitors (hair loss, muscle spasm, dysosmia, etc). The above adverse events are within the controllable range. Glasdegib is suitable for long-term treatment (158). Also, glasdegib combined with low-dose cytarabine significantly prolonged the OS of patients, and the therapeutic effect of this combination therapy was more prominent in patients with secondary AML. The third-stage clinical development of glasdegib for 7 + 3 intensive chemotherapy is underway (159).

In AML patients who could not receive chemotherapy or patients with high-risk myelodysplastic syndrome, the combined regimen of glasdegib and cytarabine is therapeutic. However, there were 12 gene mutation states which had no obvious correlation with clinical reaction. This shows that the relationship between gene mutation and response or nonresponse to treatment may not be significant, which should be further studied (160). In addition, glasdegib combined with JAKI has certain potential for MF treatment (155).

## 9 The Chiral Small Molecular Targeted Antitumor Drugs Approved by the FDA in 2019

Zanubrutinib (20), darolutamide (21), and alpelisib (22) were approved to its list by the FDA in 2019. Zanubrutinib is for adult mantle cell lymphoma (R/R MCL) patients who had received at least one treatment before. Darolutamide is for nonmetastatic castration-resistant prostate cancer (NM-CRPC). Alpelisib combined with fulvestrant is for the treatment of advanced metastatic breast cancer with hormone receptor positive (HR+)/human epidermal growth factor receptor 2 negative (HER2−) and PIK3CA mutation in male and postmenopausal women. The active enantiomers of the three drugs are (S)-enantiomers.

### 9.1 Zanubrutinib

#### 9.1.1 New Mechanisms

Zanubrutinib and ibrutinib share the same binding site as ibrutinib in BTK-cysteine 481 in the adenine triple-binding pocket of BTK, which means that with the further extension of follow-up, C481S mutant cells may be reported (161).

Ibrutinib and zanubrutinib can show different inhibitions of action in the stages of virus entry and replication, and have the potential to become new candidate drugs for inhibiting the onset of COVID-19 (162).

#### 9.1.2 New Indications

In the treatment of Waldenstrom macroglobulinemia, the incidence and severity of BTK-related toxic events after aanubrutinib treatment were lower than those of ibrutinib. In addition, the two both showed good curative effect, and the difference was not statistically significant (163). Waldenstrom macroglobulinemia has a rare complication—Bing-Neel syndrome, which is often manifested as clonal lymphoplasmacyte infiltration in the central nervous system, in which zanubrutinib has a good effect (164).

Ibrutinib and zanubrutinib showed similar inhibitory effects on MCL cell line Rec-1. Meanwhile, the inhibitory effect of zanubrutinib on ITK is 20 times lower than that of ibrutinib, and it takes 10–45 times of zanubrutinib to achieve the same inhibitory effect on PLCγ1 or IL-2 secretion as ibrutinib. The two are equally effective and more selective *in vitro*. In addition, ibrutinib showed a more significant inhibitory effect on NK cells than zanubrutinib (165). In the treatment of patients with R/R MCL, zanubrutinib achieved a high response rate of 84% in patients, and PFS was significantly prolonged. Meanwhile, good tolerance and safety were observed. Also, zanubrutinib has monotherapy activity on activated B-cell (ABC)-diffused large B-cell lymphoma cell line (161).

At the same time, BTK inhibitors can improve the symptoms of dyspnea, hypoxia, and thromboinflammation in patients with COVID-19, and the specific mechanism is still under further study (162).

#### 9.1.3 Novel Combination Regimens

In addition, zanubrutinib showed synergistic effect on all cell lines with MEK inhibitor pimasertib and BCL2 inhibitor venetoclax in ibrutinib-sensitive model (166). Zanubrutinib and BET bromide inhibitor birabresib are synergistic in three cell lines and additive in two cell lines, while XPO1 antagonist selinexor is beneficial in four cell lines (synergistic in three cell lines and additive in one) (167).

Eighty-one patients with CLL/SLL or R/R follicular lymphoma (FL) showed good overall tolerance and low incidence of adverse reactions after using the regimen of zanubrutinib and obinuzumab. The controlled trial of sample enlargement is in progress (168).

### 9.2 Darolutamide

#### 9.2.1 New Mechanisms

The results of transactivation experiments showed that darolutamide and its two optical isomers and major metabolite-keto-darolutamide showed strong competitive antagonism against AR wild type, which have differences in inhibiting AR activity in prostate cancer VCaP, LAPC-4, and LN CaP cell lines. In addition, darolutamide and its enantiomers and metabolites on AR dimerization have inhibitory activities in prostate cancer AR wild type, AR W742C mutant, and AR W742L mutant cell lines (169).

After intravenous or oral administration, the level of (S,S)-darolutamide was higher than that of (S,R)-darolutamide, and it was also observed that (S,R)-darolutamide changed to (S,S)-darolumide. The diastereomer is similar to keto-darolutamide in pharmacology *in vitro*. In addition, due to the high protein binding characteristics of keto-darolutamide, its contribution in human body is low (170).

The functional gain mutation of AR is one of the main reasons for the resistance of PCa to AR antagonists. In 68 AR mutants, darolutamide showed a complete inhibitory effect on AR mutants in 67, even if the concentration of AR mutants increased, there was no partial activation. Bicalutamide led to partial or complete activation to 63% mutants, and eight mutants were completely or partially activated by enzalutamide (171). The research preliminarily confirmed the broad-spectrum inhibitory effect of darolutamide on AR and its mutants.

Mechanically, darolutamide could block the whole genome AR enhancer, super-enhancer activation, and downstream transcription. In addition, a dynamic AR cistron dependent on androgen level was found, which exists in the high AR affinity region of prostate cancer cell lines and tissue samples. Darolutamide causes the binding of AR to genome to be greatly reduced, which strongly inhibits the activation of normal enhancer and super-enhancers, and then hinders several downstream pathways which are very important for prostate cancer proliferation. In addition, carcinogenic super-enhancers is easily affected by the defects of cellular DNA repair mechanism, which provides a theoretical basis for the effective antitumor effect of endocrine therapy combined with DNA damage repair drugs. Related clinical research is underway (172).

Interleukin-23(IL-23) can significantly inhibit the cell aging induced by enzalutamide or darolutamide in castration-resistant C4-2, and 22Rv1 cells, but not in androgen-sensitive LNCaP cells. This indicates that castration-resistant PCa cells have specific reaction to IL-23 which may be one of the causes of resistance to AR antagonists. Developing targeted drugs for IL-23 may be an effective way to improve the efficacy of AR antagonists. The specific reasons for the different expression of IL-23 in above three cells are still under further study (173).

In addition, the overexpression of AKR1C3 is the key regulator of drug resistance of castration-resistant PCa to apalutamide and darolutamide. KV-49g can significantly inhibit AKR1C3 and greatly improve the drug resistance of cancer cells to these two drugs. Therefore, AKR1C3 is a new potential target, and KV-49g is an emerging lead compound, which is currently used for preclinical evaluation (174).

#### 9.2.2 New Indications

In the nonstatistical casting-resistant prostate cancer of Japan, the efficacy and safety of darolutamide has been proved. However, it is impossible to determine whether there is a safety difference between the Japanese population and the general ARAMIS population (175).

Comparing the efficacy and safety of darolutamide, apalutamide, and enzalutamide, the three drugs can obtain the median metabolism-free survival prolongation effect superior to placebo compared with placebo, among which apalutamide has the best effect, followed by enzalutamide and darolutamide. In prostate-specific antigen progression-free survival (PSA-PFS), the performance of three drugs is the same as the median metabolism-free survival. Darolutamide has the lowest incidence of adverse events and the most controllable tolerance (176).

#### 9.2.3 Novel Combination Regimens

The combination regimens of darolutamide and other drugs provide almost no DDI, but possibly exert influence on metabolic enzymes or transporters. In addition, the inhibition of darolutamide and rosuvastatin on intestinal efflux transporters was observed, but which did not show significant impact on safety. There is little interaction between darolutamide and other compounds at therapeutic concentration, so the increased exposure of intestinal efflux transporter substrates and possible hepatic uptake transporter substrate may be the main interaction (174). The low potential DDI of darolutamide ensures less complications in the treatment of nonmetastatic castration-resistant prostate cancer.

### 9.3 Alpelisib

#### 9.3.1 New Mechanisms

Tamoxifen is the first-line hormone therapy drug for premenopausal women with estrogen receptor (ER)-positive metastatic breast cancer, and the activation of PI3K/AKT pathway will lead to resistance. Alpelisib or buparlisib and tamoxifen have synergistic effects in treating ER-positive breast cancer cell lines with different PI3K mutations. *In vitro*, significant tumor shrinkage was observed, and their synergistic effect depended on PIK3CA, AKT, and wild-type ER. Potentially, ER and PI3K/AKT pathways are similar to a bidirectional circuit, and the circuit is a standby mechanism for each other (177).

Alpelisib can inhibit the phosphorylation of AKT, mTOR, and ribosomal protein S6, while rapamycin can activate the phosphorylation of AKT (178). When the two are combined, this effect is reversed, and the overall expression is the inhibition of AKT phosphorylation, thus inhibiting the growth of tumor cells (179).

#### 9.3.2 New Indications

In the treatment of lipoma associated with PIK3CA-related overgrowth syndrome, alpelisib has shown good therapeutic effect (179).

#### 9.3.3 Novel Combination Regimens

PI3K inhibitor and tamoxifen have synergistic effect, which can also delay the occurrence of drug resistance to any single treatment (177). Alpelisib can show good antitumor activity *in vitro* and *in vivo* when used alone or in combination with paclitaxel. Meanwhile, alpelisib with paclitaxel can significantly inhibit the migration of cancer cells. The combination of these two drugs has the value of further clinical research (180).

The combination therapy of alpelisib and olaparib had good efficacy and safety in patients with epigenetic ovarian cancer, and the maximum tolerable dose was determined to be 200 mg q.d. and 200 mg b.i.d. Preliminarily, alpelisib combined with olaparib showed significant synergistic effect in ovarian cancer to platinum resistance to gBRCAwt and BRCAwt. This combination scheme superior to monotherapy has research value (181).

In nonkeratinizing nasopharyngeal carcinoma models, different results were obtained by using ribocilib alone and using ribocilib and alpelisib in combination. Combined medication significantly inhibited tumor growth, and the tumor volume decreased even more (182). *In vitro*, when alpelisib is used alone or in combination with rapamycin, the reduction of cell proliferation is characterized by concentration and time dependence. Alpelisib alone did not directly kill cells but accelerated cell aging (179).

In colorectal cancer patients with BRAF mutation, double therapy (encorafenib 200 mg/day + cetuximab) and triple therapy (encorafenib 200 mg/day + alpelisib 300 mg/day + cetuximab) showed good clinical efficacy and safety. At the same time, it was observed that alpelisib in triple-therapy group had mild DDI with encorafenib at higher dose, that is, the exposure of encorafenib increased by 2 times. This may be because alpelisib inhibited CYP3A4, the metabolic enzyme of encorafenib. Cetuximab and encorafenib did not affect the exposure of alpelisib (117).

## 10 Discussion

The introduction of the concept of chirality provides new ideas for the research of small molecular targeted anticancer drugs. Most of the chiral small molecular targeted drugs approved by the FDA are the single enantiomer, and the enantiomers of these drugs are either inactive, low in activity, or high in activity. Crizotinib in market, for example, has a chiral compound with the (R)-configuration, approved by the FDA. Although the (S)-configuration is not approved, it targets different enzyme named MTH1, making it certain anticancer activity either. The (R)-configuration of ruxolitinib shows higher target binding priority than the (S)-configuration because of its stereostructure specificity. Darolutamide and its (S,S)-configuration and (S,R)-configuration as well as its metabolites have AR inhibitory activity *in vivo*.

In this review, the chiral small molecular targeted drugs approved by the FDA from 2011 to 2019 were systematically summarized, and the new mechanisms, new indications, and novel combination regimens of each drug in recent years were presented. It can be seen that the research on unapproved enantiomers of these drugs is relatively scarce, and researchers pay less attention to explore the chiral characteristics of these drugs, which may be directly related to the difficulty of chiral resolution in certain situations. In fact, the chirality of drug molecules will directly affect its distribution, absorption, metabolism, and excretion *in vivo*, as well as pharmacodynamics and pharmacotoxicology. A well-designed study of chiral drug molecules can better grasp the metabolism and mechanism of drug molecules in human body (178).

Undeniably, small molecular targeted drugs have certain limitations in the treatment of tumors. Many patients often need genetic testing to determine whether they meet the medication requirements. Drug resistance and relatively high treatment cost also become the limitations of further popularization of this treatment method. Clinically, small molecular targeted drugs are often used in combination with other drugs to achieve the purpose of optimizing efficacy, overcoming drug resistance, and improving safety. In addition, it is a pleasure for researchers to actively explore new indications of existing small molecular targeted drugs in order to expand the population of beneficiary patients, and the research results of some drugs have been satisfactory. Talazorib was initially approved by the FDA for the treatment of breast cancer with BRCA gene mutation. Because of its remarkable curative effect, researchers actively carried out research on other indications of this drug, and finally found that talazoparib had unexpected therapeutic effect on triple negative breast cancer, and this indication has been approved by the FDA (183, 184).

We have speculated that the reason that talazoparib shows distinctive superiority is probably related to its chirality. As the fourth-generation PARP inhibitor, its efficacy is more than 100 times that of the first-generation PARP inhibitor olaparib (no chirality), which may also be caused by its chiral center. Therefore, in order to effectively solve the current problems in small molecular targeted therapy, the advantage of chiral structure to drugs is a kind of guidance, which will lead us to find a new idea for directing our furture research.

In a word, studying the pharmacological activities of drug molecules and their chiral enantiomers can provide extensive data for the clinical application of drugs, help researchers to know the role of chiral centers in drug activity, and provide new evidence and ideas for the joint use of drugs and the discovery of new signal pathways.

## Author Contributions

XC: data curation and writing—original draft preparation. YB: software. XY: writing—reviewing and editing.

## Funding

This work was supported by the National Natural Science Foundation of China (No. 81874212, 82172653); Huxiang High-Level Talent Innovation Team (2018RS3072); Opening Fund for Key Laboratory of Molecular Pharmacology and Drug Evaluation (Yantai University), Ministry of Education (P201905); Scientific and Technological Projects for Collaborative Prevention and Control of Birth Defect in Hunan Province (2019SK1012); and Key Grant of Research and Development in Hunan Province (2020DK2002).

## Conflict of Interest

The authors declare that the research was conducted in the absence of any commercial or financial relationships that could be construed as a potential conflict of interest.

## Publisher’s Note

All claims expressed in this article are solely those of the authors and do not necessarily represent those of their affiliated organizations, or those of the publisher, the editors and the reviewers. Any product that may be evaluated in this article, or claim that may be made by its manufacturer, is not guaranteed or endorsed by the publisher.
